# Supplementary material for: Melting Curve Analysis of Aptachains: Adenosine Detection with Internal Calibration
Source: Biosensors (Basel). 2021 Apr 8;11(4):112. doi: 10.3390/bios11040112 (PMC8068264; doi:10.3390/bios11040112)
Supplement: Supplementary file 1 [file biosensors-11-00112-s001.pdf]

Supplementary Information:

Melting curve analysis of Aptachains: Adenosine detection with internal calibration

Chenze Lu, Christine Saint-Pierre, Didier Gasparutto, Yoann Roupioz, Corinne Ravelet, Eric Peyrin, Arnaud Buhot

Temperature Scans

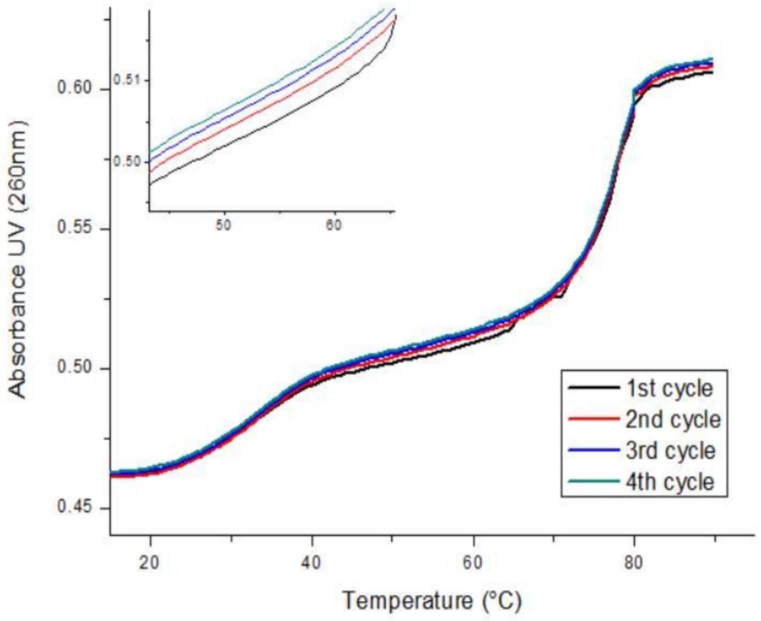

Fig.S1: Experimental data of UV absorbance at 260 nm for four consecutive scans with SA3-6A/B dimers. The two melting points are clearly visible and lead to two melting peaks in the melting curves (derivative of the UV absorbance). A slight evaporation is observed with an increase of the absorbance upon the consecutive cycles (inset from 45 to 65°C).

Gel electrophoresis experiments

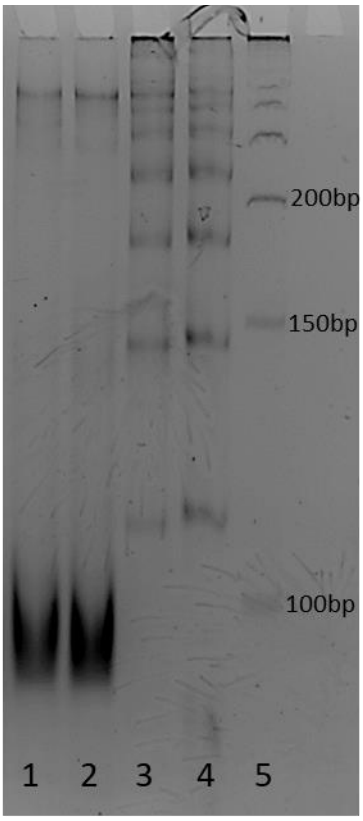

Fig. S2: Electrophoresis results on acrylamide gels 10%. Lines 1 and 2 were the SA5-6A/SA5-6B dimers with 1mM of adenosine in the gel, lines 3 and 4 were the fully commentary SA5-6A/SA5-6Ac dimers. Line 5 was the DNA ladder for indicating the length of the structure.

The lines 1 and 2 presented a broad band corresponding to the length of the SA5-6A/SA5-6B dimers with hybridization of the complementary red strands (see Table below). No aptamer bridges were present while the concentration of adenosine in the gel was set at a large value (1 mM). On the contrary, the lines 3 and 4 presented several bands corresponding to the binding of multiple dimers through the self-complementarity of the aptamer bridges hybridization (blue part of SA5-6Ac complementary to the split-aptamer in SA5-6A). Those electrophoresis experiments suggested that the 1D chains with adenosine bridges were not strong enough to support migration through acrylamide gel even in presence of a large amount of adenosine (1 mM). However, when self-complementary strands were considered the chain formation of various lengths were present (several bands).

Name	Sequence 5’ to 3’
SA5-6A	**CCTGCGGAGGAAGGTTCGACCATCGTGCGGGTAGGTAGA**
SA5-6B	**GAACCTGGGGGAGTAGGTCTACCTACCCGCACGATGGTC**
SA5-6Ac	**GAACCTTCCTCCGCAGGTCTACCTACCCGCACGATGGTC**
